# The relationship between food insecurity, purchasing patterns and perceptions of the food environment in urban slums in Ibadan, Nigeria

**DOI:** 10.1186/s40795-024-00929-8

**Published:** 2024-09-18

**Authors:** Temitope Ilori, Nicola Christofides, Laurel Baldwin-Ragaven

**Affiliations:** 1 Family Medicine Unit, Department of Community Medicine, Faculty of Clinical Sciences, Ibadan, Nigeria; 2 Department of Family Medicine, University of Ibadan/University College Hospital, Ibadan, Nigeria; 3 School of Public Health, University of the Witwatersrand, Johannesburg, South Africa; 4 Department of Family Medicine and Primary Care, School of Clinical Medicine, Faculty of Health Sciences, University of the Witwatersrand, Johannesburg, South Africa

**Keywords:** Food insecurity, Food environment, Coping strategies, Purchasing patterns, Urban slum, Ibadan

## Abstract

**Background:**

Rapid urbanisation without concomitant infrastructure development has led to the creation of urban slums throughout sub-Saharan Africa. People living in urban slums are particularly vulnerable to food insecurity due to the lack of physical and economic accessibility to food. Hence, it is important to explore how vulnerable groups living in slums interact with the food environment. This study assessed the relationships between food insecurity, including restrictive coping strategies, food purchasing patterns and perceptions about the food environment among dwellers of selected urban slums in Ibadan, Nigeria.

**Methods:**

This community-based cross-sectional study was conducted with people responsible for food procurement from 590 randomly selected households in two urban slums in Ibadan. Food insecurity and restrictive coping strategies were assessed using the Household Food Insecurity Access Scale and the Coping Strategy Index, respectively. We examined purchasing patterns of participants by assessing the procurement of household foodstuffs in different categories, as well as by vendor type. Participants’ perceptions of the food environment were derived through a five-item composite score measuring food availability, affordability and quality. Chi-square tests and logistic regression models analysed associations between food insecurity, purchasing patterns and perceptions of the food environment.

**Results:**

The prevalence of food insecurity in the sample was 88%, with 40.2% of the households experiencing severe food insecurity. Nearly a third (32.5%) of the households used restrictive coping strategies such as limiting the size of food portions at mealtimes, while 28.8% reduced the frequency of their daily meals. Participants purchased food multiple times a week, primarily from formal and informal food markets rather than from wholesalers and supermarkets. Only a few households grew food or had livestock (3.2%). Food insecure households had a lower perceived access to the food environment, with an approximate 10% increase in access score per one-unit decrease in food insecurity (AOR = 0.90, 95% CI: 0.84, 0.96). The most procured foods among all households were fish (72.5%), bread (60.3%), rice (56.3%), yam and cassava flours (50.2%). Food-secure households procured fruit, dairy and vegetable proteins more frequently.

**Conclusion:**

Food insecurity remains a serious public health challenge in the urban slums of Ibadan. Perceptions of greater access to the food environment was associated with increasing food security. Interventions should focus on creating more robust social and financial protections, with efforts to improve livelihoods to ensure food security among urban slum-dwellers.

**Supplementary Information:**

The online version contains supplementary material available at 10.1186/s40795-024-00929-8.

## Introduction

Urbanisation is one of the most significant environmental changes that has reshaped the world in the last two centuries [1, 2]. Approximately 55% of the world’s population currently lives in urban areas, and this figure is expected to rise to 60% in 2030 and 68% by 2050 [3]. Rapid urbanisation in low- and middle-income countries (LMICs) has occurred without concomitant infrastructure development, resulting in urban slums [2]. According to United Nations Habitat, one-third of people in urban areas in LMICs live in slums [3]. However, in Africa, 62% of the total population live in slums [4]. The explosion of urban slums particularly in LMICs creates many challenges, including overcrowding, lack of essential services, substandard housing, restricted access to healthcare, unsafe water and inadequate nutrition and sanitation [5]. Some of the nutritional vulnerabilities are due to insufficient food supply as well as the high cost of food staples and the low purchasing abilities of the residents [6–8], putting them at a greater risk of food insecurity [9].

Food security is a social determinant of health and a sustainable development goal (SDG). Goal 2 of the 2030 Agenda for Sustainable Development set by the United Nations in 2015 aims to “eradicate hunger” and “ensure access by all people, including the poor and vulnerable people, to safe, nutritious and sufficient food all year round” (SDG Target 2.1) and to “eradicate all forms of malnutrition by 2030” (SDG Target 2.2) [10]. Food insecurity was defined at the 1996 World Food Summit as “the lack of physical, social and economic access to sufficient, safe and nutritious food for an active and healthy life” [11]. Food insecurity currently affects 10% of the global population; however, one-third of people living in Africa are burdened with severe food insecurity [12]. A study by the African Food Security Urban Network (AFSUN) revealed that only 17% of African households were food secure, while 57% to 70% were severely food insecure [13]. In 2021, 70% of Africans experienced moderate to severe food insecurity [14]. In 2014, almost 85% of slum residents in Nairobi, Kenya were food insecure, with 50% being severely food insecure [9]. Similarly, an 81% prevalence of food insecurity was found among 230 sampled urban slum dwellers in Ibadan, Nigeria in 2018 [15].

Generally, people living with food insecurity have an increased risk of developing different adverse health outcomes [16]. A systematic review and meta-analysis of non-communicable diseases (NCDs) modifiable by diet in sub-Saharan Africa showed a compelling association of household food insecurity with key metabolic risk factors, such as malnutrition, overweight, obesity, dyslipidaemia, hypertension, underweight and micronutrient deficiencies [16]. Individuals with food insecurity tend to purchase and consume low-cost, less varied, nutrient-poor and calorie-dense foods, making them vulnerable to the double burden of malnutrition and NCDs [17]. Children living in food-insecure households are frequently stunted and malnourished [18, 19].

Members of households who cannot access enough food employ specific behavioural coping strategies to adapt to food insufficiency. For example, some may make restrictive alterations of the food they consume, while might engage in socially unacceptable, negative or shameful activities [20]. Such behaviours include reducing the quantity and quality of food consumed, rationing food among family members according to different sentiments or privileges at the expense of others, begging, skipping meals due to a lack of money or borrowing from friends and neighbours. These behavioural responses are proxy indicators for household food access and food insecurity [21].

The food environment is defined as the physical and social interface between the consumer and the food system that encompasses the availability, affordability, convenience and desirability of different foods items [22]. Urbanisation and the nutrition transition in LMICs are changing the food environment and influencing household food access. Food sources impact household diet quality, nutrition and health [17]. In urban Africa, traditional open markets remain the primary sources for food purchasing [23]. However, the incursion of supermarkets and hypermarkets into the urban food environment has led to a higher intake of ultra-processed and lower-quality foods [24–26]. Food procurement is influenced by food availability and access, and eating a healthy diet is difficult without access to healthy foods [23, 25]. Food purchasing patterns provide insights into household dietary intake; however, these are influenced by a complex interplay between nutrition literacy, personal preferences and the food environment [27]. Appelhans et al. demonstrated that household food purchases are an unbiased estimate of household diet quality, although they are less accurate for determining specific nutrient intake [28]. Unable to produce most of the food they consume, urban dwellers are less self-sufficient than those living in rural environments as they are dependent on food purchasing [29]. Therefore, their diet quality is more dependent on fluctuating food prices and changes in the global food environment [30].

Although access to food is a basic human right, people living in urban slums are at a higher risk of inadequate access to quality and highly nutritious food item due to lack of physical and economic access to buy healthy food [31]. Despite the high prevalence of food insecurity across urban cities in sub-Saharan Africa [9, 13, 32], very few studies have been conducted among vulnerable slum dwellers in Nigeria [15, 33]. Therefore, it is essential to explore more deeply the perceptions and interactions of these households with the food environment to provide recommendations for relevant interventions to improve access to healthy food options and address food security in these communities. Hence, this study aimed to assess the relationship between food insecurity, including restrictive coping strategies, and household purchasing patterns and perceptions of access to the food environment in two selected urban slums in Ibadan, Southwest Nigeria.

## Materials and methods

### Study design and setting

This cross-sectional study was conducted in two urban slums of Ibadan, Oyo State, Nigeria. Ibadan is the capital of Oyo State, located in the southwest geo-political zone of Nigeria. Ibadan, the most populous city in Oyo State is home to twelve identifiable urban slums due to rapid unplanned urbanisation [34]. Two of these slums, Idikan and Sasa, were selected as the study sites as they are typical of inner-city slums and because of an existing relationship with the University of Ibadan that includes a data collection infrastructure. All structures in both study sites were geo-mapped using satellite imagery for an earlier study [35, 36]. The two slums are located approximately seven kilometres (km) from each other. Idikan is a low-income traditional community located in the city core. It has 1617 households and a population of 13,000 inhabitants [37]. The inhabitants are mostly Yoruba, the main ethnic group in Oyo State, and predominantly traders and artisans. In contrast, Sasa is a fourth generation resettled migrant community inhabited primarily by ethnic Hausa–Fulani Muslims from northern Nigeria. It is located on the city’s periphery. It has 1755 households, with a population of approximately 8,000 inhabitants. The two slums are characterised by high poverty rates, poor housing, limited infrastructure such as non-potable water and problematic waste disposal, high levels of violence and insecurity, unemployment and poor health indicators [35].

### Sampling and participant recruitment

All residential dwellings in each slum formed the sampling frame for the survey. The street listings were used to draw a random list of streets, after which computer-generated random numbers were used to select individual houses per street until the sample size 590 was reached. If there was more than one household within the same residential dwelling, one was randomly selected for sampling. The adult most responsible for household food purchases was chosen as the informant in each identified household. Any eligible respondent who could not be found at home was revisited at least twice before moving to the next dwelling house to the right of the selected house. Six dwellings were replaced when no one in that household could be contacted.

### Sample size

The sample size was determined using the Leslie Kish formula *N* = Z^2^pq/d^2^ [38] where *N* = sample size; Z = confidence level (which was taken as 95% with a degree of probability of 1.96%); *p* = total prevalence of food insecurity taken as 85.0% [9]; q = 1.0 – p; and, d^2^ = level of precision, assumed to be 3%. A total of *N* = 544 was thus calculated, and an anticipated nonresponse rate of 10% was added (+ 54). The final sample of 590 households was recruited from the two study sites. With a sample size of 590, this study has a power of 90% to detect a significant difference in perceptions about the accessibility of the food environment between food-secure and severely food-insecure participants (at a significance level of 5%).

### Data collection

A total of 590 respondents, who were mainly responsible for food procurement, were interviewed face-to-face by a single research assistant using a structured questionnaire (the survey instrument). Data were captured on Android tablet devices with Open Data Kit (ODK) software. ODK is a mobile technology developed at the University of Washington that permits offline data collection and uploads all submissions once the device is connected to the internet [39]. The first author (TI) recruited four postgraduate master’s students in Public Health at the University of Ibadan as data collectors for this study. They underwent training on the objectives of the study, communication skills, obtaining informed consent, administering the questionnaires, taking anthropometric measurements and maintaining ethical standards. The training also included capturing data using the software and uploading data onto the server after collection.

### Survey instrument

The questionnaire consisted of respondents’ sociodemographic and household characteristics, the Household Food Insecurity Access Scale (HFIAS) [40] and the Coping Strategies Index (CSI) [21]. The HFIAS and CSI are validated instruments used in other African countries, including Nigeria, with good internal consistency [7, 41, 42]. In addition, the survey instrument also assessed patterns of household food procurement and perceptions about the accessibility of the food environment in the slum settings. Lastly, measures of the respondents’ body mass index (BMI) were recorded. The investigators developed the questionnaire following an extensive literature review and previous practice experience [26, 43].

The instrument was translated to Yoruba and Hausa, the commonly spoken languages in the study sites and back translated to English to ensure the original meanings of the questions were retained. Feedback from the pilot study were used to modify the questionnaires to eliminate response ambiguity.

### Measures

The outcome variable for this study was the household food insecurity score assessed using the HFIAS developed by Food and Nutrition Technical Assistance (FANTA) and funded by United States Agency for International Development (USAID) [40]. Nine frequency-of-occurrence questions measured both food security and three gradients of food insecurity over the past 30 days. Possible responses to each question included whether the condition occurred rarely (once or twice), sometimes (three to ten times) or often (more than ten times). A Likert scale of ‘never' (a score of 0), 'rarely' (scored 1),'sometimes' (scored 2) and 'often' (scored 3) was then assigned and summed. The lowest possible score was 0, indicating that a household is food secure, with the highest score of 27, indicating a severely food-insecure household. The cut scores for the four categories are 0–1 (food-secure), mildly food insecure (2–7), moderately food insecure (8–14) and severely food insecure [44]. In this sample, the Cronbach’s alpha for the HFIAS was 0.94. While we report all four categories, for analysis purposes, the mildly food insecure households were merged with food secure households and re-categorized as food secure due to the small frequency of both categories. Hence, only three categories were used for further statistical analysis: ‘food secure’ representing the initial ‘food secure’ and ‘mildly insecure’ categories; ‘moderately’ food insecure; and ‘severely food insecure’. In the regression analyses, however, responses were dichotomized into either food-secure or food-insecure households.

Household food coping behaviours were assessed using the Coping Strategies Index (CSI) [21], a proxy indicator of food insecurity. The CSI contains questions on how members of the household respond to food inadequacies. The CSI was calculated using information on how often a household used a set of eleven food-based coping strategies in the last 30 days. The frequency of occurrences (the relative frequency categories) was a measure of how many days in a week a household had to rely on the various restrictive coping strategies, ranging from “never” to “every day”. The possible responses for each of the eleven coping strategies (frequency of occurrences) in a week were as follows: ‘never’; ‘hardly at all’ (< 1 day per week); ‘once in a while’ (1–2 days per week); ‘pretty often’ (3–6 days per week) and ‘daily’ and were scored according to the midpoint value of the range of each category. Based on the community context, we weighted certain behaviours according to their social undesirability. For example, the strategy to “send household members to beg” scored an 8, while “rely on less preferred and less expensive foods” weighed 2 points. The more undesirable a behaviour, the higher the assigned weighting score. The composite score was then calculated as the frequency with which each coping strategy is used multiplied by its severity weight. The weighted product summation of the coping strategies gives the CSI composite score, which was subsequently categorized into low (0–50), medium (51–100) and high (over 100). The Cronbach’s alpha of the CSI in this sample was 0.85.

### Explanatory Variables

#### Availability, affordability and quality: a composite score of participants’ perceptions of the food environment

Access to food in the respondents’ food environment was assessed through the perceived availability, affordability and quality of food choices. We created a five-item self-report questionnaire for this study from the relevant literature [26, 43] to determine the perception of accessibility to food in the community. The first author drafted the initial pool of questions to assess accessibility to the food environment. This initial pool of questions was reviewed and assessed for face validity by the other two authors, academic scholars with public health expertise. Accessibility to the food environment was gauged by asking respondents about their ability to “do most of their food shopping at stores close to their house”; “if the food markets in their neighbourhood offer a wide variety of food items”; and, if the food products sold in their neighbourhood “are usually fresh” and are “sold at lowest selling price”. Finally, we inquired about “food vendors selling prepared foods”. We created a scoring system using a 5-point Likert scale, with responses of “Strongly Disagree”, “Disagree”, “Neutral”, “Agree” or “Strongly Agree” scored from 1 to 5. After summing these responses, a “composite score of the perceptions of the food environment” was reported, with a maximum possible score of 25 indicating highly positive perceptions of the food environment, with the lowest possible score of 5. Exploratory Factor Analysis (EFA) was used to validate the underlying constructs of the perceptions of the food environment scale that was created for this study. Principal components analysis was used because the primary purpose was to identify and compute composite scores for the factors underlying the accessibility of the food environment. Eigenvalues showed that one factor accounted for 42% of the variance and that there were no other factors. Items in this analysis had factor loadings over 0.30 with four having factor loadings above 0.50 (Supplementary Table 1). The composite score of the five items had a mean of 18.1 with a SD of 3.2. The skewness was -0.27 and the kurtosis was 3.7. The internal consistency was satisfactory, with a Cronbach’s alpha of 0.66.

The food purchasing patterns of households were assessed by inquiring about what foods were bought from the different food groups (grains, roots and tubers, legumes, vegetables, fruits, animal protein, dairy products, and ultra-processed foods) adapted from the dietary diversity index [40]. The frequency of purchase was also assessed, with responses ranging from never (< 1 per week) to sometimes (1–2 per week) and often (3–7 times per week). The tool also assessed from which type of vendors (formal food market, informal food market, supermarkets, and wholesalers) purchases were made.

The food outlets were described as follows: i) formal market: a public open-air market where food is sold by local vendors; ii) informal market: street vendors that sell a small selection of food and other goods; iii) supermarket: a large store selling a variety of food and household items at retail prices; and, iv) wholesale store: stores that sell different categories of food items at wholesale prices.

### Sociodemographic characteristics

Sociodemographic characteristics of the respondent, who was the person most responsible for food procurement in the household, included age, gender, educational status, marital status and type (monogamous/polygamous), household size, money spent on food weekly and wealth index. The household wealth index was calculated using principal component analysis from information collected about housing quality (floors and wall material), type of cooking fuel and ownership of modern household assets using the 11-item Equity Tool. The Equity Tool is a country-specific wealth index variable based on the Demographic and Health Survey [45]. Questions such as: “What is the main material of the walls and floors in your house?”; “Does your household have electricity, fan, television, refrigerator, generating set, or cable TV?”; “What type of fuel does your household mainly use for cooking?”; and “Does any member of your household have a bank account?” were asked. The Equity Tool was scored into five quintiles. However, due to small cell numbers, we merged these five categories into three for analysis: the lowest category being “poorer”, the middle category being “moderate”, and the highest category being “wealthier”.

### Body Mass Index (BMI)

BMI was calculated using body weight (kg)/height (m^2^). The weight and height of each respondent were measured according to standard protocols. Weight was measured in 0.1 kg by use of the Omron® electronic bathroom weighing scale. Height was measured to the nearest 0.1 m using a stadiometer. Cut points of < 18.50, 25.00–29.99, and > 30 kg/m^2^ were used for underweight, overweight and obesity, respectively. Normal BMI was between 18.5 and 24.9 kg/m^2^.

### Data analysis

After coding, data were entered, cleaned and analysed using STATA Version 15.0 software (Stata Corp., College Station, TX, USA). The sociodemographic characteristics of respondents and households within each food (in)security category were described using frequencies and percentages. Descriptive statistics were used for categorical variables such as food sources and purchasing patterns, while means and standard deviations (SDs) were used to summarise perceived perceptions of the food environment scores. Chi-square tests determined the relationship between the coping strategies of food-secure and food-insecure households and assessed the relationships between HFIAS and sociodemographic characteristics. One-way ANOVA was used to determine the association between HFIAS, and perceptions of the food environment scores, and post hoc analysis (Bonferroni) was used to assess the distribution of the scores across the three levels of food insecurity. A binary logistic regression analysis model was built to assess the adjusted odds ratio (AOR) and 95% confidence interval (CI) for the association between HFIAS status and perceived access to the food environment while controlling for sociodemographic variables that were statistically significant in the univariate analyses. Multivariate ordinal logistic regression was used to model the relationship between the frequency of food purchased across the three levels of food security. The level of significance for all analyses was set with a *p*-value less than 0.05.

## Results

A total of 590 adults who were most responsible for purchasing food for their households were interviewed. This indicated a response rate of 100%, which is not uncommon in this study setting [33]. Approximately one-third (35%) of the respondents were household heads, while 60.7% were the spouses of household heads. Table 1 summarises the sociodemographic characteristics of the respondents and their households. Approximately 85% of respondents were between the ages of 18 and 59, with an average age of 42.3 ± 14.4 years. Most respondents (88.3%) were women, and 73.4% were married. Seventy-two per cent (72.0%) of the married respondents were in monogamous marriages. The mean household size was 4.8 ± 2.4, with approximately one-third (30.4%) of households falling into the poorer wealth index. Almost two-thirds (59.7%) of respondents had completed secondary school. The majority (80.7%) of the household heads were engaged in paid manual labour. Most respondents (83.9%) earned less than ₦50,000 per month (equivalent to USD131 at the time of the study) [46]; and 50.2% of respondents spent less than ₦5000 (USD13) weekly on food. The mean BMI of participants was 24.2 (SD ± 5.4), with 10% of the sample underweight and 11.9% obese, suggesting the presence of the double burden of malnutrition.

**Table 1:** Socio-demographic characteristics of the respondents

**Variables**	**n (%)**
**Age**	
18 – 29	125 (21.2)
30 – 39	143 (24.2)
40 – 49	120 (20.3)
50 – 59	116 (19.7)
60 and above	86 (14.6)
**Sex**	
Male	69 (11.7)
Female	521 (88.3)
**Marital Status**	
Single / Never married	48 (8.1)
Married	433 (73.4)
Once married	109 (18.5)
**Type of marriage (*n* = 418)**	
Monogamous	301 (72.0)
Polygamous	117 (28.0)
**Level of education**	
No Formal Education	88 (14.9)
Primary	150 (25.4)
Secondary and above	352 (59.7)
**Household size**	
1 – 3	176 (29.8)
4 – 7	349 (59.2)
8 and above	65 (11.0)
**Weekly food expenditure (*n* = 522)**	
Less than ₦5000 (< $13)*	262 (50.2)
> ₦5000 < 10000 (> $13 < $26)*	179 (34.3)
> ₦10000 (> $26) *	81 (15.5)
**Wealth Index**	
Poorer	178 (30.4)
Moderate	199 (34.0)
Wealthier	209 (35.6)
**Location**	
Slum A	293 (49.7)
Slum B	297 (50.3)
**BMI**	
Less than 18.5	59 (10.0)
18.5 – 24.9	315 (53.4)
25.0 – 29.9	146 (24.7)
30.0 and above	70 (11.9)

^*^Currency conversion on 15 May 2021: Naira 380.5 = 1 USD [46]

### Prevalence of household food insecurity

**Fig. 1:**
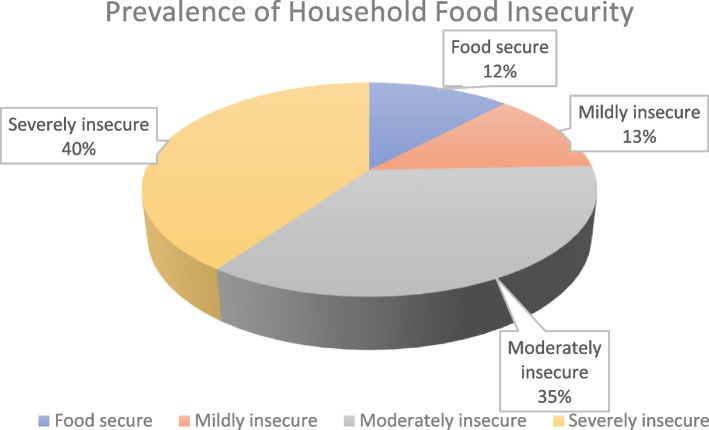
Prevalence of household food insecurity as measured by the HFIAS

Findings from this study revealed that only 12% of the surveyed households were food secure. All the other households experienced some degree of food insecurity, with 40.2% experiencing severe food insecurity, 35.4% experiencing moderate food insecurity and 12.4% experiencing mild food insecurity (Fig. 1).

### Restrictive coping strategies

**Fig. 2:**
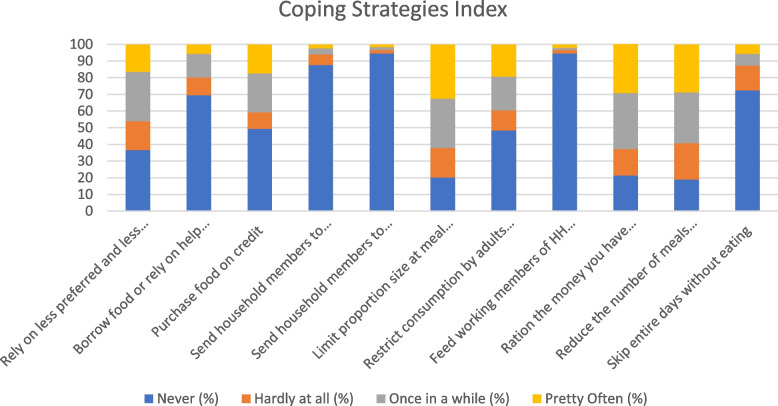
Coping strategies index

The three most common restrictive coping strategies to deal with food insecurity were to limit portion size at mealtimes (32.5%), ration the money at hand, buy prepared food (29.3%) and reduce the number of meals eaten in a day (28.8%). Adults also limited their consumption so that small children could eat (19.3%). Other coping strategies, such as skipping entire day without eating (5.6%), borrowing food or relying on friends or relatives (5.6%), sending household members to eat elsewhere (2.4%) and begging (1.7%), were practised less often (Fig. 2).

### Association between food insecurity and respondents’ perceptions of the food environment: availability, affordability and quality of food

Table 2 demonstrates that favourable perceived accessibility to the food environment was significantly associated with greater food security. The food-secure households had higher composite scores (mean ± SD) of perceptions of the food environment (18.94 ± 3.9) than the moderately (17.9 ± 3.0) and severely (17.8 ± 2.8) food-insecure (*p* = 0.001). A significantly higher proportion of the respondents (*n* = 527; 89.3%), agreed that the food markets in their neighbourhood offered a wide variety of food items. Additionally, 86.8% (*n* = 512) of the respondents agreed that they could do most of their food shopping near their homes. Most respondents agreed that the food products sold in their neighbourhood were usually fresh (*n* = 489; 82.9%), with higher proportions of food-secure respondents (*n* = 125; 86.8%) agreeing with this item, compared to 81.8% (*n* = 171) and 81.4% (*n* = 193) of moderately and severely food-insecure respondents, respectively. Nearly two-thirds of the respondents (*n* = 380; 64.4%) disagreed that foods were sold at the lowest selling price, which was endorsed more by the severely food insecure respondents (*n* = 172; 72.6%) (*p* < 0.001). The vast majority of respondents (*n* = 536; 90.8%) agreed that there were many options for food vendors selling prepared foods. Household food production, whether growing vegetables (*p* = 0.95) or keeping livestock (*p* = 0.99), was not significantly associated with food security. Food-insecure households were less likely to own a refrigerator; moreover, owning a refrigerator was significantly associated with food security (*p* =  < 0.001) (Table 2).

**Table 2:** Association between household food insecurity and respondents’ perceptions of accessibility of the food environment and quality of food

**Variable**	**Total** **(*N*** = 590) **n (%)**	**Food Security Status**			***p*-value**
		**Food secure** **(*n*** = 144) **n (%)**	**Moderately insecure** **(*n*** = 209) **n (%)**	**Severely insecure** **(*n*** = 237) **n (%)**	
**Composite score of perceptions of the food environment (mean ± SD)**	18.13 (3.2)	18.94 (3.9)	17.9 (3.0)	17.8 (2.8)	0.001**
**I can do most of my food shopping at shops close to me**					
Disagree	57 (9.7)	18 (12.5)	25 (12.0)	14 (5.9)	0.02*
Neutral	21 (3.6)	6 (4.2)	11 (5.3)	4 (1.7)	
Agree	512 (86.8)	120 (83.3)	173 (82.8)	219 (92.4)	
**The food markets in my neighbourhood offer a wide varieties of food items**					
Disagree	24 (4.1)	10 (6.9)	7 (3.3)	7 (3.0)	0.32
Neutral	39 (6.6)	11 (7.6)	12 (5.7)	16 (6.8)	
Agree	527 (89.3)	123 (85.4)	190 (90.9)	214 (90.3)	
**The food products sold in my neighbourhood are usually fresh**					
Disagree	70 (11.9)	9(6.2)	27(12.9)	34(14.3)	0.14
Neutral	31(5.3)	10(6.9)	11(5.3)	10(4.2)	
Agree	489(82.9)	125(86.8)	171(81.8)	193(81.4)	
**Foods are sold at lowest selling price**					
Disagree	380(64.4)	73(50.7)	135(64.6)	172(72.6)	< 0.001**
Neutral	45(7.6)	23(16.0)	13(6.2)	9(3.8)	
Agree	165(28.0)	48(33.3)	61(29.2)	56(23.6)	
**There are lots of options of food vendors selling prepared foods**					
Disagree	27(4.6)	6(4.2)	8(3.8)	13(5.5)	0.17
Neutral	27(4.6)	10(6.9)	12(5.7)	5(2.1)	
Agree	536(90.8)	128(88.9)	189(90.4)	219(92.4)	
**Autonomous food production and storage**					
**Grow vegetables for food**					
Yes	19(3.2)	5(3.5)	7(3.3)	7(3.0)	0.95
No	571(96.8)	139(96.5)	202(96.7)	230(97.0)	
**Keep livestock for food**					
Yes	154(26.1)	37(25.7)	55(26.3)	62(26.2)	0.99
No	436(73.9)	107(74.3)	154(73.7)	175(73.8)	
**Own a refrigerator**					
Yes	180(30.5)	66(45.8)	59(28.2)	55(23.2)	< 0.001**
No	410(69.5)	78(54.2)	150(71.8)	182(76.8)	

^*^p < 0.05, **p < 0.01

One-way ANOVA showed a statistically significant difference in the perceived food environment across the three levels of food insecurity (F = 6.039, *p* = 0.003). Post hoc analysis (Bonferroni) showed that food-secure participants have a significantly higher perception of the food environment than moderately food-insecure (*p* = 0.09) and severely food-insecure participants (*p* = 0.04). However, there was no statistical difference in the perceptions of the food environment between moderately and severely food-insecure participants (*p* = 1.00) (Table 3).

**Table 3:** Post-hoc analysis of the relationship between food security and the perception of the accessibility of the food environment

**(I) Food security**	**(J) Food security**	**Mean Difference (I-J)**	***p*-value**
**Food secure / Mildly insecure**	Moderately Insecure	1.04	0.009**
	Severely insecure	1.09	0.004**
**Moderately Insecure**	Food secure / Mildly insecure	-1.04	0.009**
	Severely insecure	0.05	1.000
**Severely insecure**	Food secure / Mildly insecure	-1.09	0.004**
	Moderately Insecure	-0.05	1.000

^**^p < 0.01

### Factors associated with household food insecurity following logistic regression analysis

Food insecurity was significantly associated with being age 40–59 years, having only primary education, larger household size (eight members and above) and not owning a refrigerator (*p* = 0.001) (Table 4). Respondents at different food insecurity levels did not differ significantly by gender (*p* = 0.40) or marital status (*p* = 0.3) nor by location (*p* = 0.150).

**Table 4:** Univariate and multivariate analysis of food insecurity in households and the composite perception of the food environment score

**Variable**	**Unadjusted odds ratio (OR)**	**95% confidence interval**	***p*-value**	**Adjusted OR**	**95% CI**	***p*-value**
**Composite accessibility score**	1.11	1.05, 1.18	0.001*	0.90	0.84; 0.96	0.002**
**Sex**						
Male	Ref			Ref		
Female	1.306	0.70, 2.43	0.398	1.49	0.76; 2.88	0.24
**Age**						
18 – 29	Ref			Ref		
30 – 39	0.796	0.47, 1.34	0.391	1.06	0.59; 1.91	0.84
40 – 49	0.385	0.21, 0.71	0.002*	2.32	1.16; 4.67	0.02*
50 – 59	0.401	0.22, 0.74	0.004*	2.05	1.02; 4.12	0.04*
60 and above	0.840	0.46, 1.52	0.566	1.08	0.53; 2.22	0.83
**Level of education**						
No Formal Education	Ref			Ref		
Primary	0.349	0.18, 0.66	0.001*	4.51	2.14; 9.51	< 0.001**
Secondary and above	0.792	0.48, 1.31	0.367	3.62	1.79; 7.31	< 0.001**
**Household size**						
1–3	Ref			Ref		
4–7	0.684	0.41, 1.13	0.137	1.47	0.90; 2.42	0.12
8 and above	0.254	0.12, 0.54	< 0.01*	5.81	2.04; 16.54	0.001**
**Marital status**						
Single/never married	Ref			Ref		
Married	0.687	0.36, 1.31	0.256	0.62	0.31; 1.25	0.19
Once married	0.689	0.32, 1.46	0.332	0.65	0.29; 1.45	0.29
**Location**						
Slum A	Ref			Ref		
Slum B	1.319	0.90, 1.92	0.150	1.27	0.87; 1.87	0.22
**Owning a refrigerator**						
Yes	Ref					
No	0.41	0.27, 0.60	0.001*	1.69	1.99; 2.87	0.05*

While adjusting for sociodemographic characteristics; *p < 0.05, **p < 0.01; Ref = reference

In the multiple regression model (Table 4), households that were food insecure had lower perceived access to the food environment, with an approximately 10% increase in access score per one-unit decrease in food insecurity (AOR = 0.90 (95% CI: 0.84, 0.96)). Respondents who did not own refrigerators had higher odds of being food insecure (AOR = 1.69 (95% CI: 1.99, 2.87). Respondents aged 40–49 had an adjusted OR = 2.32 (95% CI: 1.16, 4.67) and those 50–59 had an adjusted OR = 2.05 (95% CI: 1.02, 4.12) compared to those 18–29 years, doubling the odds of being food insecure.

Respondents with primary education were four times more likely to be food insecure than those without formal education (AOR = 4.45 (95% CI: 2.14, 9.51)). The odds of food insecurity among households with large family sizes (≥ 8) were nearly six times higher than those of households with fewer than three family members (AOR = 5.81 (95% CI = 2.04, 16.54)).

### Food purchasing behaviours

Table 5 indicates that there was a high reliance on traditional formal food markets (58.6%) and informal street markets (45.1%) for daily food procurement, with only approximately 1.2% of respondents purchasing food items from supermarkets often. Less than 10% of respondents frequently sourced their foods from wholesalers, indicating an inability to buy in bulk.

**Table 5:** Frequency of purchase from different types of food vendors

	**Frequency of purchase**		
**Vendor type**	** < 1** × **week n (%)**	**1–2** × **week n (%)**	**3–7 × week n (%)**
Formal food market	59 (10.0)	185 (31.4)	346 (58.6)
Informal food market	77 (13.0)	247 (41.9)	266 (45.1)
Wholesalers	411 (69.7)	123 (20.9)	56 (9.4)
Supermarket	543 (92.0)	40 (6.8)	7 (1.2)

### Association between household food insecurity and frequency of types of food purchased

Table 6 shows that processed carbohydrates such as white rice and bread (61.7%), animal proteins (72.9%), vegetables (66.8%) and fruits (60.2%) were the most frequently purchased items by households across all levels of food security. Starchy staples such as yam and cassava and their flours (21.9%), legumes such as beans and nuts (57.8%) and milk and milk products (46.8%) were purchased slightly less frequently (weekly/monthly). Although food-secure households purchased complex carbohydrates (roots and tubers), vegetable proteins (beans and nuts), fruits, leafy vegetables and milk and dairy products more frequently, ultra-processed foods such as sweets, ice cream, and sugar-sweetened beverages were purchased rarely.

**Table 6:** Association between household food insecurity and frequency of types of food purchased

**Variable**	**Total (*N*** = 590)	**Food security Status**			**X**^**2**^** / F**	***p*-value**
		**Food secure (*N*** = 144)	**Moderately Insecure (*N*** = 209)	**Severely Insecure (*N*** = 237)		
**Processed grains (white rice/bread)**	n (%)	n (%)	n (%)	n (%)		
Daily	101 (17.1)	16 (11.1)	30 (14.4)	55(23.2)	43.85	< 0.001**
Weekly	364 (61.7)	79 (54.9)	134 (64.1)	151 (63.7)		
Monthly	108 (18.3)	47 (32.6)	41 (19.6)	20 (8.4)		
Rarely	17 (2.9)	2 (1.4)	4 (1.91)	11 (4.64)		
**Unprocessed/ minimally processed starchy roots, tubers and flours)**						
Daily	29 (4.9)	4 (2.8)	8 (3.8)	17 (7.17)	28.21	< 0.001**
Weekly	129 (21.9)	30 (20.8)	51 (24.4)	48 (20.2)		
Monthly	142 (24.1)	54 (37.5)	50 (23.9)	38 (16.03)		
Rarely	290 (49.1)	56 (38.9)	100 (47.9)	134 (56.5)		
**Vegetable Proteins (Legumes; beans, nuts)**						
Daily	66 (11.2)	10 (6.9)	29 (13.9)	27 (11.39)	14.62	0.023*
Weekly	341 (57.8)	80 (55.6)	128 (61.2)	133 (56.1)		
Monthly	119 (20.2)	41(28.5)	34 (16.3)	44 (18.6)		
Rarely	64 (10.8)	13 (9.0)	18 (8.61)	33 (13.9)		
**Animal proteins (meat, chicken, fish)**						
Daily	137 (23.2)	33 (22.9)	52 (24.9)	52 (21.9)	4.44	0.618
Weekly	430 (72.9)	105 (72.9)	148 (70.8)	177 (74.7)		
Monthly	17 (2.9)	5 (3.47)	8 (3.8)	4 (1.7)		
Rarely	6 (1.0)	1 (0.7)	1 (0.5)	4 (1.7)		
**Fruits (bananas, avocados, oranges, tangerines)**						
Daily	109 (18.5)	40 (27.8)	32 (15.3)	37 (15.6)	27.98	< 0.001**
Weekly	355 (60.2)	87 (60.4)	136 (65.1)	132 (55.7)		
Monthly	76 (12.9)	13 (9.0)	28 (13.4)	35 (14.77)		
Rarely	50 (8.5)	4 (2.8)	13 (6.2)	33 (13.9)		
**Vegetables (broccoli, okra, pumpkin)**						
Daily	147 (24.9)	35 (24.3)	47 (22.5)	65 (27.4)	13.34	0.038*
Weekly	394 (66.8)	91 (63.2)	148 (70.8)	155 (65.4)		
Monthly	37 (6.3)	16 (11.1)	12 (5.7)	9 (3.8)		
Rarely	12 (2.0)	2 (1.4)	2 (1.0)	8 (3.4)		
**Milk and dairy products**						
Daily	31 (5.2)	10 (6.9)	12 (5.74)	9 (3.8)	28.89	< 0.001**
Weekly	276 (46.8)	86 (59.7)	95 (45.4)	95 (40.1)		
Monthly	118 (20.0)	29 (20.14)	45 (21 .5)	44 (18.6)		
Rarely	165 (28.0)	19 (13.2)	57 (27.8)	89 (37.5)		
**Ultra-processed (cakes, sweets and chocolate)**						
Daily	50 (8.5)	16 (11.1)	16 (7.7)	18 (7.6)	43.13	< 0 .001**
Weekly	176 (29.8)	66 (45.8)	66 (31.6)	44 (18.6)		
Monthly	46 (7.8)	13 (34.0)	17 (8.1)	16 (6.8)		
Rarely	318 (53.9)	49 (34.0)	110 (52.6)	159 (67.1)		
**Ultra-processed (ice cream, soft drinks)**						
Daily	33 (5.6)	13 (9.0)	10 (4.8)	10 (4.2)	62.97	< 0.001**
Weekly	142 (24.1)	61 (42.4)	56 (26.8)	25 (10.5)		
Monthly	142 (24.1)	12 (8.3)	56 (26.8)	25 (10.5)		
Rarely	369 (62.5)	58 (40.3)	127 (60.8)	184 (77.6)		
**Beverages (tea, coffee)**						
Daily	64 (10.8)	24 (16.7)	23 (11.0)	17 (7.2)	52.08	< 0.001**
Weekly	240 (40.7)	73 (50.7)	96 (45.9)	71 (30.0)		
Monthly	87 (14.8)	25 (17.4)	29 (13.9)	33 (13.9)		
Rarely	199 (33.7)	22 (15.3)	61 (29.2)	116 (49.0)		

^*^p < 0.05, **p < 0.01

### Multivariate ordinal logistic regression analysis of the relationship between types of food purchased and food insecurity

In the multivariate ordinal logistic regression (Table 7), monthly purchase of processed grains was associated with five times lower odds of food insecurity than daily purchase (OR: (95% CI: 0.16 – 0.75). Monthly (OR: 2.48 (95% CI: 1.28 – 4.78)) and rarely (OR: 3.74 (95% CI: 1.74 – 8.06)) purchasing of fruits and vegetables were also significantly associated with higher odds of food insecurity compared to daily. However, there are nearly two times higher odds of food insecurity amongst those who purchased animal protein weekly than those who purchased it daily (OR: 1.76 (95% CI: 1.13 – 2.73). Rare purchase of ultra-processed foods was significantly associated with higher odds of food insecurity (OR: 2.19 (95% CI: 1.18 – 4.08)).

**Table 7:** Multivariate ordinal logistic regression analysis of the relationship between types of food purchased and food insecurity

**Variable**	**Odds Ratio (OR) (95% CI)**	***p*-value**
**Processed grains (white rice/bread)**		
Daily	Reference	
Weekly	0.647 (0.380–1.100)	0.108
Monthly	0.267 (0.140—0.512)	< 0.01**
Rarely	1.130 (0.360–3.549)	0.834
**Unprocessed/minimally processed starchy roots, tubers and flours**		
Daily	Reference	
Weekly	0.587 (0.216–1.592)	0.295
Monthly	0.497 (0.182–1.356)	0.172
Rarely	0.951(0.373–2.425)	0.916
**Vegetable proteins: legumes, beans or nuts**		
Daily	Reference	
Weekly	0.894 (0.503–1.590)	0.703
Monthly	0.763 (0.385–1.514)	0.439
Rarely	0.542 (0.252 -1.167)	0.118
**Animal proteins: meat, chicken or fish**		
Daily	Reference	
Weekly	1.757 (1.133–2.725)	0.012*
Monthly	0.872 (0.316–2.407)	0.792
Rarely	1.858 (0.278–12.402)	0.523
**Fruits and vegetables**		
Daily	Reference	
Weekly	1.514 (0.887–2.584)	0.129
Monthly	2.476 (1.282–4.781)	0.007**
Rarely	3.741(1.736–8.061)	0.001**
**Ultra-processed foods**		
Daily	Reference	
Weekly	0.746 (0.395–1.412)	0.369
Monthly	1.210 (0.551–2.657)	0.635
Rarely	2.190 (1.175- 4.083)	0.014*

^*^p < 0.05, **p < 0.01

## Discussion

There were high levels of food insecurity among households in two urban slums in Ibadan, with 40.2% experiencing severe food insecurity. This was also reflected in the adoption of restrictive coping strategies by participants. Households with poorer perceptions of the food environment, including the availability, affordability and quality of food, were more likely to experience food insecurity in this study. Most households frequently purchased traditional staples such as rice, yam, cassava flour, beans and other legumes regardless of their level of food insecurity. However, food-insecure households relied more heavily on processed carbohydrates such as rice and bread and bought these foods frequently (daily or weekly). Food-secure households were significantly more likely to purchase fruits, vegetables and dairy products, often daily. The majority of households rarely purchased ultra-processed foods such as sugar-sweetened drinks, sweets or ice cream but food secure households did so more often than food insecure households.

The high prevalence of food insecurity observed in this study is consistent with previous studies in Africa [9, 15, 47, 48] and strikingly similar to the 81% food insecurity prevalence found in urban slum households in Ibadan, Nigeria, which also employed the HFIAS [15]. Furthermore, maladaptive coping strategies were employed to manage limited access to food. The restrictive coping strategies most commonly used were reducing portion sizes at mealtimes and reducing the number of meals eaten in a day. Households with severe food insecurity adopted more stigmatised coping strategies such as sending household members to eat elsewhere or beg for food. These patterns were similar to studies conducted in Bangladesh and Ethiopia [49, 50].

Our study showed that few households had vegetable gardens or kept livestock. Urban slums may not be conducive to maintaining gardens, as was reported in urban South Africa, where only 2.3% of low-income households grew their own food [13]. Traditionally, residents of Ibadan urban slums were farmers. However, as urban dwellers they no longer engaged in growing food due to limited arable land and instead depended on purchasing food to meet their dietary needs [33]. A systematic review by Warren et al. assessed the association between urban agriculture and food insecurity with mixed results [51]. The review reported that some studies found that households with home gardens were able to prevent hunger, increase dietary diversity and reduce rates of childhood stunting; however, others did not report these associations [51].

Most household food security was a function of the purchasing abilities of the residents in our study, with formal and informal markets closest to the home being the primary sources of food procurement. Few residents shopped in supermarkets or in wholesale shops. Our study revealed that households with positive perceptions of the food environment were less likely to be food insecure. The moderate and severe food insecure households perceived neighbourhood food markets as not very affordable with a lower quality of food. Previous studies showed that regular acquisition of minimally processed foods was associated with the perceived availability of fresh produce in the neighbourhood [52, 53]. Similarly, a systematic review of the local food environment and diet revealed that households that reported easy access to supermarkets consumed more portions of fruits and vegetables than those with poorer access [27]. Similar studies documented an association between perceived food access and consumption of fruits and vegetables [42, 54].

Chen et al., in a systematic review of 20 epidemiological studies in 2020, concluded that there was a strong association between the consumption of ultra-processed foods and cardiovascular diseases [55]. In the slum settings in Ibadan, ultra-processed food such as ice cream and soft drinks were purchased infrequently but food secure households purchased these products to a greater extent that food insecure households. This pattern may become more pronounced with marketing as has been seen in other settings [56]. Future research in these settings needs to explore the effects of food marketing especially of processed and ultra-processed food to better understand this phenomenon.

Although both food-secure and food-insecure households frequently purchased traditional staple foods such as maize, cassava and yams as well as processed grains such as rice and bread, food-insecure households bought processed carbohydrates (rice and bread) more often. Research from Mozambique, the Philippines and Canada [57–60] similarly revealed how families that are unable to grow their own food and have limited income to purchase food are likely to opt for the cheapest cost per calorie from the available choices. In our study, food-secure households were significantly more likely to purchase dairy products, a variety of fresh vegetables and fruits and did so more often. This suggests that with greater relative affluence, there is greater food diversity. Of potential concern, however, is that food secure households purchased ultra-processed foods more commonly suggesting a possible shift in patterns that have been seen elsewhere. Similar shifts were observed in urban areas of some LMICs that are undergoing a nutrition transition [57–60]. Recent evidence suggests that these changes in dietary patterns result in rising levels of obesity among people experiencing poverty with attendant adverse health implications [61].

Traditional food staples such as maize, cassava and yams remain rich sources of essential micronutrients, are low in glycaemic index, high in dietary fibre and protective against gastrointestinal cancers [62]. Evidence from a systematic review and meta-analysis indicated that a higher dietary fibre intake was associated with a 15–30% reduction in mortality from cardiovascular diseases (CVD), and a reduced incidence of NCDs and colorectal cancers [54]. These traditional staples are also low in saturated fat, sugar and salt, reducing the risk of cardiovascular diseases [63, 64].

Severely food insecure households shopped more frequently which could result in a vicious cycle of poverty because so much time is spent purchasing food daily, leaving little time to engage in income-generating activities to improve food security. Although a regular supply of electricity is a challenge in Nigeria [65], ownership of refrigerators was protective against food insecurity in the study areas. Half of the food-secure households had refrigerators, enabling them to buy and store perishable foods such as vegetables and fruits, thereby improving access. These patterns were similar to those reported by Spieker et.al in seven slum sites across Nigeria, Kenya, Pakistan and Bangladesh [33].

Many African cities are undergoing major shifts in the food environment with the emergence of supermarkets selling a wide variety of food products in urban areas to consumers [13, 66]. However, these food stores are usually located in higher-income areas and are not within the reach of many urban poor [67, 68]. As observed in this study, very few urban slum dwellers shopped in supermarkets. Although one study from Kenya found supermarkets to be sources of healthy food options [24], other studies in a systematic review revealed that frequent exposure to supermarkets may increase the consumption of ultra-processed foods [27, 28]. Living in residential areas with more supermarkets and fast food restaurants close to households was significantly associated with increased intake of unhealthy food in Australia [69]. However, due to the limited access to these supermarkets in our study, many residents relied on smaller formal and informal markets for their household food needs. These informal markets sell traditional local food staples and fresh healthy food choices such as meat, fish, fruits and vegetables. Although a study in Vietnam expressed concern about the safety and quality of foods sold in informal food markets [25], they remain a major food source in urban slums because of their availability and easy accessibility [67]. These markets and informal stores allow shoppers to bargain and offer credit facilities to familiar customers while operating flexibly, which in South Africa sometimes involved opening for extended hours [70].

### Strengths and limitations of this research

The strength of this research was to provide insights into food purchasing behaviours across different levels of food insecurity in urban slums as well as highlight perceptions of the food environment. However, this study was cross-sectional, thereby limiting the ability to investigate temporal relationships and draw causal inferences. Since the survey data were collected via self-reported measures, it is possible that there was recall bias. Additionally, respondents could have given socially acceptable responses or underreported socially undesirable events. In this study, participants were asked questions on the frequency of purchases without measuring the quantities of food bought or consumed. The measure developed to assess perceptions of the food environment should ideally be validated in future studies with different populations.

### Recommendations

This research could be used to inform a variety of interventions to improve patterns of food purchasing in urban slum environments. Food-insecure households less frequently purchased plant-based proteins. Vegetable-based proteins are cheaper, with lower-caloric density and less fats and cholesterol, providing a healthy alternative to animal proteins and should be encouraged among the residents.

Household income in this study was low. Local government authorities could implement social protection mechanisms and initiatives to improve livelihoods. Households in slum communities could be supported to strengthen their collective purchasing capabilities, affording them healthier diets. Local governments could also make land available for communal food gardens, including agricultural cultivation education and supplies, and offer incentives to purchase traditional staple foods such as maize, yams and cassava for household consumption. Communal solar-powered cold storage facilities for preservation could also be provided to improve community food security. Health workers should organise nutritional education for residents on the benefits of consuming nutritious, minimally processed traditional staple foods.

## Conclusion

Household food insecurity is exceptionally high among urban slum dwellers in Ibadan, Nigeria. Many households adopted negative or restrictive coping strategies in response to food insecurity. Households with better perceptions of the food environment were more likely to be food secure. The food purchasing patterns of these urban slum households were shaped by their levels of food insecurity. Food-secure households were more likely to purchase more traditional nutrient-dense, minimally processed foods, including fruits and vegetables, while food-insecure households purchase more processed foods. However, the purchase of ultra-processed foods was significantly associated with food-secure households. It is important to preserve the consumption of healthy traditional foods in sufficient quantity in these resource-constrained communities through a range of household-based and food environment interventions supporting healthier food choices. Food security is a basic human right; and, as such, local governments have an obligation to protect these rights and ensure that all people have access to safe and sufficient food.

## Supplementary Information

Supplementary Material 1. 

## Data Availability

The datasets are available from the corresponding author on reasonable request.

## Abbreviations

AOR: Adjusted Odds Ratio
CVD: Cardiovascular Diseases
CBN: Central Bank of Nigeria
CI: Confidence interval
CSI: Coping Strategy Index
FANTA: Food and Nutrition Technical Assistance
FAO: Food and Agriculture Organization
HFIAS: Household Food Insecurity Access Scale
IRB: Institutional Review Board
LMICs: Low- and Middle-Income Countries
NCDs: Non-communicable Diseases
SDG: Sustainable Development Goals
WHO: World Health Organisation

## Data availability

No datasets were generated or analysed during the current study.
