# Supplementary material for: The relationship between food insecurity, purchasing patterns and perceptions of the food environment in urban slums in Ibadan, Nigeria
Source: BMC Nutr. 2024 Sep 18;10:122. doi: 10.1186/s40795-024-00929-8 (PMC11409710; doi:10.1186/s40795-024-00929-8)
Supplement: Supplementary file 1 — Supplementary Material 1. [file 40795_2024_929_MOESM1_ESM.pdf]

Supplementary table 1: Factor loadings based on a principal components analysis with a varimax rotation for five items (n=590)

Item	Factor1
You can do most of your food shopping at shops/stores close to your house	0.6319
The food markets in your neighbourhood offer a wide variety of food items for sale.	0.7526
The food products sold in your neighbourhood are usually fresh	0.5245
Foods are sold at the lowest selling price in your neighbourhood.	0.3796
There are lots of options of food vendors selling prepared/cooked foods close to where you live.	0.5099
